# Supplementary material for: The association between shift work exposure and cognitive impairment among middle-aged and older adults: Results from the Canadian Longitudinal Study on Aging (CLSA)
Source: PLoS One. 2023 Aug 23;18(8):e0289718. doi: 10.1371/journal.pone.0289718 (PMC10446236; doi:10.1371/journal.pone.0289718)
Supplement: S1 Checklist — (DOCX) [file pone.0289718.s001.docx]

STROBE Statement—checklist of items that should be included in reports of observational studies

|  | Item No. | Recommendation | Reported on Page  No. |
| --- | --- | --- | --- |
| **Title and abstract** | 1 | (*a*) Indicate the study’s design with a commonly used term in the title or the abstract | Page 2: Abstract: methods |
|  |  | (*b*) Provide in the abstract an informative and balanced summary of what was done and what was found | Page 2-3: Abstract: background, methods, results, conclusion |
| Introduction | | | |
| Background/rationale | 2 | Explain the scientific background and rationale for the investigation being reported | Page 4-6 : Introduction: paragraphs 1-4 |
| Objectives | 3 | State specific objectives, including any prespecified hypotheses | Page 7: Introduction: paragraph 5 |
| Methods | | | |
| Study design | 4 | Present key elements of study design early in the paper | Page 7: Materials and methods: Study design and sample: paragraphs 1-2 |
| Setting | 5 | Describe the setting, locations, and relevant dates, including periods of recruitment, exposure, follow-up, and data collection | Page 7: Materials and methods: Study design and sample: paragraphs 1-2 |
| Participants | 6 | (*a*) *Cohort study*—Give the eligibility criteria, and the sources and methods of selection of participants. Describe methods of follow-up  *Case-control study*—Give the eligibility criteria, and the sources and methods of case ascertainment and control selection. Give the rationale for the choice of cases and controls  ***Cross-sectional study*—Give the eligibility criteria, and the sources and methods of selection of participants**  (*b*) *Cohort study*—For matched studies, give matching criteria and number of exposed and unexposed  *Case-control study*—For matched studies, give matching criteria and the number of controls per case | ***Cross-sectional study***  Page 7: Materials and methods: Study design and sample: paragraphs 1-2 |
| Variables | 7 | Clearly define all outcomes, exposures, predictors, potential confounders, and effect modifiers. Give diagnostic criteria, if applicable | Page 8-11: Materials and methods:  Assessment of primary exposure:paragraphs1-4  Primary outcome: paragraphs 1-3, Table 1  Potential predictors: paragraph 1 |
| Data sources/ measurement | 8* | For each variable of interest, give sources of data and details of methods of assessment (measurement). Describe comparability of assessment methods if there is more than one group | Page 8-11: Materials and methods:  Assessment of primary exposure:paragraphs1-4  Primary outcome: paragraphs 1-3, Table 1  Potential predictors: paragraph 1 |
| Bias | 9 | Describe any efforts to address potential sources of bias | Page 11: Materials and methods:  Analysis: paragraph 1  Page 12: Results: paragraph 1 |
| Study size | 10 | Explain how the study size was arrived at | N/A  No sample size was calculated as this was a secondary data analysis from CLSA data base. The CLSA provides survey weights (i.e., inverse probability weights) and analytical weights, which were used for prevalence estimates and regression modelling respectively to generalize results to the Canadian population. |

Continued on next page

| Quantitative variables | 11 | Explain how quantitative variables were handled in the analyses. If applicable, describe which groupings were chosen and why | Page 11: Materials and methods:  Analysis: paragraph 1 |
| --- | --- | --- | --- |
| Statistical methods | 12 | (*a*) Describe all statistical methods, including those used to control for confounding  (*b*) Describe any methods used to examine subgroups and interactions  (*c*) Explain how missing data were addressed  (*d*) *Cohort study*—If applicable, explain how loss to follow-up was addressed  *Case-control study*—If applicable, explain how matching of cases and controls was addressed  *Cross-sectional study*—If applicable, describe analytical methods taking account of sampling strategy  (*e*) Describe any sensitivity analyses | Page 11: Materials and methods:  Analysis: paragraph 1  Page 12: Results: paragraph 1: Comparisons of complete versus missing cases  Supplementary data (S1 Appendix) |
| Results | | | |
| Participants | 13* | (a) Report numbers of individuals at each stage of study—eg numbers potentially eligible, examined for eligibility, confirmed eligible, included in the study, completing follow-up, and analysed  (b) Give reasons for non-participation at each stage  (c) Consider use of a flow diagram | Page 7: Materials and methods:  Study design and sample: paragraphs 1-2  Page 8: Figure 1 (participant flowchart ) |
| Descriptive data | 14* | (a) Give characteristics of study participants (eg demographic, clinical, social) and information on exposures and potential confounders  (b) Indicate number of participants with missing data for each variable of interest  (c) *Cohort study*—Summarise follow-up time (eg, average and total amount) | Page 12-15: Results: paragraph 2  Table 2 (summary statistics) |
| Outcome data | 15* | *Cohort study*—Report numbers of outcome events or summary measures over time  *Case-control study—*Report numbers in each exposure category, or summary measures of exposure  ***Cross-sectional study—*Report numbers of outcome events or summary measures** | Page 12-15: Results: paragraphs 2-3  Table 2 (summary statistics) |
| Main results | 16 | (*a*) Give unadjusted estimates and, if applicable, confounder-adjusted estimates and their precision (eg, 95% confidence interval). Make clear which confounders were adjusted for and why they were included  (*b*) Report category boundaries when continuous variables were categorized  (*c*) If relevant, consider translating estimates of relative risk into absolute risk for a meaningful time period | Page 15-18: Result: paragraphs 4-9  Table 3 and 4 |

Continued on next page

| Other analyses | 17 | Report other analyses done—eg analyses of subgroups and interactions, and sensitivity analyses | Page 12: Results: Paragraph 1: Comparisons of complete versus missing cases  Supplementary data (S1 Appendix) |
| --- | --- | --- | --- |
| Discussion | | | |
| Key results | 18 | Summarise key results with reference to study objectives | Page 19: Discussion: paragraph 1 |
| Limitations | 19 | Discuss limitations of the study, taking into account sources of potential bias or imprecision. Discuss both direction and magnitude of any potential bias | Page 22-23: Discussion: paragraph 7 |
| Interpretation | 20 | Give a cautious overall interpretation of results considering objectives, limitations, multiplicity of analyses, results from similar studies, and other relevant evidence | Page 19-22: Discussion: paragraph 2-6 |
| Generalisability | 21 | Discuss the generalisability (external validity) of the study results | Page 22-23: Discussion: paragraph 7 |
| Other information | |  | |
| Funding | 22 | Give the source of funding and the role of the funders for the present study and, if applicable, for the original study on which the present article is based | Page 23: Acknowledgement |

*Give information separately for cases and controls in case-control studies and, if applicable, for exposed and unexposed groups in cohort and cross-sectional studies.

**Note:** An Explanation and Elaboration article discusses each checklist item and gives methodological background and published examples of transparent reporting. The STROBE checklist is best used in conjunction with this article (freely available on the Web sites of PLoS Medicine at http://www.plosmedicine.org/, Annals of Internal Medicine at http://www.annals.org/, and Epidemiology at http://www.epidem.com/). Information on the STROBE Initiative is available at www.strobe-statement.org.
