# Supplementary material for: The association between shift work exposure and cognitive impairment among middle-aged and older adults: Results from the Canadian Longitudinal Study on Aging (CLSA)
Source: PLoS One. 2023 Aug 23;18(8):e0289718. doi: 10.1371/journal.pone.0289718 (PMC10446236; doi:10.1371/journal.pone.0289718)
Supplement: S1 Appendix — (DOCX) [file pone.0289718.s002.docx]

**S1 Appendix**

**Comparisons of complete versus missing cases.**

| **Comparison of baseline properties of cases included for analysis (N=47,811) versus cases with missing information related to shift work exposure (N=1,682)** | | | | |
| --- | --- | --- | --- | --- |
| **Baseline characteristics** | **Cases included (N=47811)**  **N (%)^a^** | **Missing cases**  **(N=1682)**  **N (%)^a^** | **Statistics^b^** | **P-value** |
| **Age (years)** |  |  | 12.83 | <0.05 |
| 45-54 | 12,623 (26.41) | 407 (24.20) |  |  |
| 55-64 | 15,427 (32.27) | 507 (30.14) |  |  |
| 65-74 | 11,141 (23.31) | 428 (25.45) |  |  |
| 75+ | 8,610 (18.01) | 340 (20.21) |  |  |
| **Sex** |  |  | 31.79 | <0.05 |
| Male | 23,461 (49.07) | 943 (56.06) |  |  |
| Female | 24,350 (50.93) | 739 (43.94) |  |  |
| **Ethnicity** |  |  | 0.002 | 0.96 |
| Whites | 45,784 (95.87) | 1,610 (95.89) |  |  |
| Other^c^ | 1,974 (4.13) | 69 (4.13) |  |  |
| **Marital status** |  |  | 1.16 | 0.28 |
| With partner | 32,930 (68.90) | 1,138 (67.66) |  |  |
| No partner | 14,867 (31.10) | 25.95 (32.34) |  |  |
| **Education level** |  |  | 17.79 | <0.05 |
| Less than high school | 3,759 (7.87) | 174 (10.36) |  |  |
| High school to some college | 24,564 (51.40) | 792 (47.14) |  |  |
| Bachelor’s degree and Higher | 19,467 (40.73) | 714 (42.50) |  |  |
| **Household income (CAD)** |  |  | 12.40 | 0.10 |
| Less than $20,000 | 2,567 (5.73) | 128 (8.09) |  |  |
| $20,000 or more, but less than $50,000 | 11,233 (25.09) | 435 (27.48) |  |  |
| $50,000 or more, but less than $100,000 | 16,101 (35.96) | 511 (32.28) |  |  |
| $100,000 and more | 14,869 (33.21) | 509 (32.15) |  |  |
| **Smoking** |  |  | 0.40 | 0.81 |
| Never | 14,667 (30.76) | 520 (30.99) |  |  |
| Former | 28,511 (59.79 | 1,007 (60.01) |  |  |
| Current | 4,510 (9.46) | 151 (9.00) |  |  |
| **Alcohol consumption** |  |  | 23.12 | <0.05 |
| Never | 6,946 (14.53) | 294 (17.51) |  |  |
| Drinks less than weekly | 14,607 (30.56) | 501 (29.84) |  |  |
| Drinks at least weekly | 26,237 (54.90) | 884 (52.65) |  |  |
| **BMI (kg/m^2^)** |  |  | 3.13 | 0.37 |
| 20.0-24.99 (normal weight) | 14,056 (29.65) | 473 (28.43) |  |  |
| <20.00 (underweight) | 1,466 (3.09) | 57 (3.43) |  |  |
| 25.0-29.99 (overweight) | 18,945 (38.96) | 653 (39.24) |  |  |
| >30.0 (obese) | 12,938 (27.29) | 481 (28.91) |  |  |
| **Depression** |  |  | 0.076 | 0.78 |
| No (CES-D10 <10) | 39,901 (83.46) | 1,408 (83.46) |  |  |
| Yes (CES-D10 ≥ 10) | 7,910 (16.54) | 274 (16.29) |  |  |
| **Multi-morbidity** |  |  | 3.10 | 0.08 |
| Yes (≥2 chronic diseases) | 14,363 (30.04) | 539 (32.05) |  |  |
| No (<2 chronic disease) | 33,448 (69.96) | 1,143 (67.95) |  |  |
| **Social Support Availability (SSA)** |  |  | 0.54 | 0.76 |
| Low | 16,396 (35.47) | 573 (36.20) |  |  |
| Medium | 15,148 (32.77) | 520 (32.85) |  |  |
| High | 14,681 (31.76) | 490 (30.95) |  |  |
| **Retirement status** |  |  | 0.90 | 0.34 |
| Completely/partially retired | 26,940 (56.51) | 927 (55.34) |  |  |
| Not retired | 20,729 (43.49) | 748 (44.66) |  |  |
| **Type of study cohort** |  |  | 49.09 | <0.05 |
| Tracking | 19,515 (40.82) | 543 (32.28) |  |  |
| Comprehensive | 28,296 (59.18) | 1,139 (67.72) |  |  |
| **REYI impairment** |  |  | 0.03 | 0.86 |
| No | 42,594 (94.18) | 1,502 (94.29) |  |  |
| Yes | 2,630 (5.82) | 91 (5.71) |  |  |
| **REYII impairment** |  |  | 10.61 | 0.06 |
| No | 42,382 (94.56) | 1,520 (96.45) |  |  |
| Yes | 2,436 (5.44) | 56 (3.55) |  |  |
| **AF2 impairment** |  |  | 3.94 | 0.06 |
| No | 42,501 (94.54) | 1,529 (95.68) |  |  |
| Yes | 2,456 (5.46) | 69 (4.32) |  |  |
| **MAT impairment** |  |  | 0.054 | 0.82 |
| No | 39,619 (91.92) | 1,391 (91.75) |  |  |
| Yes | 3,482 (8.08) | 125 (8.85) |  |  |
| **Overall impairment** |  |  | 1.02 | 0.31 |
| No | 39,092 (96.22) | 1,392 (96.73) |  |  |
| Yes | 1,536 (3.78) | 47 (3.76) |  |  |
| **^a^** Reported frequencies are column percentages  **^b^** The statistic reported for all variables was the chi-square value  **^c^** Other included South Asian, Chinese, Filipino, Latin American, Japanese, Southeast Asian, Korean, Arab, West Asian, and Black.  CAD, Canadian dollars; BMI, body mass index; CES-D, the Center for Epidemiological Studies Depression Scale SW, shift work; REY, Rey auditory verbal learning; AF, Animal fluency; MAT, Mental alteration. | | | | |

| **Comparison of baseline properties of complete cases of cognitive impairment variables included for analysis (N=46,610) versus cases with missing information (N=1,201)** | | | | |
| --- | --- | --- | --- | --- |
| **Baseline characteristics** | **Cases included (N=46,610)**  **N (%)^a^** | **Missing cases**  **(N=1,201)**  **N (%)^a^** | **Statistics ^b^** | **P-value** |
| **Age (years)** |  |  | 44.76 | <0.05 |
| 45-54 | 12,377 (26.55) | 246 (20.65) |  |  |
| 55-64 | 15,071 (32.33) | 356 (29.89) |  |  |
| 65-74 | 10,839 (23.25) | 302 (25.36) |  |  |
| 75+ | 8,323(17.86) | 287 (24.10) |  |  |
| **Sex** |  |  | 0.83 | 0.36 |
| Male | 22,856 (49.04) | 605 (50.37) |  |  |
| Female | 23,754 (50.96) | 596 (49.63) |  |  |
| **Ethnicity** |  |  | 8.80 | 0.16 |
| Whites | 44,656 (95.91) | 1,128 (95.87) |  |  |
| Other^c^ | 1,905 (4.09) | 69 (5.76) |  |  |
| **Marital status** |  |  | 23.28 | <0.05 |
| With partner | 32,179 (69.06) | 751 (62.53) |  |  |
| No partner | 14,417 (30.94) | 450 (37.47) |  |  |
| **Education level** |  |  | 47.69 | <0.05 |
| Less than high school | 3,614 (7.75) | 145 (12.29) |  |  |
| High school to some college | 23,924 (51.33) | 640 (54.24) |  |  |
| Bachelor’s degree and Higher | 19,072 (40.92) | 395 (33.47) |  |  |
| **Household income (CAD)** |  |  | 46.17 | <0.05 |
| Less than $20,000 | 2,476 (5.66) | 91 (8.63) |  |  |
| $20,000 or more, but less than $50,000 | 10,914 (24.97) | 319 (30.27) |  |  |
| $50,000 or more, but less than $100,000 | 15,727 (35.98) | 374 (35.48) |  |  |
| $100,000 and more | 14,599 (33.40) | 270 (25.62) |  |  |
| **Smoking** |  |  | 2.38 | 0.31 |
| Never | 14,314 (30.79) | 353 (29.42) |  |  |
| Former | 27,791 (59.78) | 720 (60) |  |  |
| Current | 4,383 (9.43) | 127 (10.58) |  |  |
| **Alcohol consumption** |  |  | 21.21 | <0.05 |
| Never | 6,724 (14.43) | 222 (18.52) |  |  |
| Drinks less than weekly | 14,222 (30.53) | 385 (32.11) |  |  |
| Drinks at least weekly | 25,645 (55.04) | 592 (49.37) |  |  |
| **BMI (kg/m^2^)** |  |  | 7.24 | 0.07 |
| 20.0-24.99 (normal weight) | 13,735 (29.71) | 321 (27.30) |  |  |
| <20.00 (underweight) | 1,417 (3.07) | 49 (4.17) |  |  |
| 25.0-29.99 (overweight) | 18,472 (39.96) | 473 (40.22) |  |  |
| >30.0 (obese) | 18,472 (27.27) | 333 (28.32) |  |  |
| **Depression** |  |  | 10.55 | <0.05 |
| No (CES-D10 <10) | 38,940 (83.54) | 961 (80.02) |  |  |
| Yes (CES-D10 ≥ 10) | 7,670 (16.46) | 240 (19.98) |  |  |
| **Multi-morbidity** |  |  | 5.04 | <0.05 |
| Yes (≥2 chronic diseases) | 13,967 (29.97) | 396 (32.97) |  |  |
| No (<2 chronic disease) | 32,643 (70.03) | 805 (67.03) |  |  |
| **Social Support Availability (SSA)** |  |  | 10.19 | <0.05 |
| Low | 15,949 (35.36) | 447 (39.98) |  |  |
| Medium | 14,806 (32.82) | 342 (30.59) |  |  |
| High | 14,352 (31.82) | 329 (29.43) |  |  |
| **Retirement status** |  |  | 28.19 | <0.05 |
| Completely /partially retired | 26,173 (56.32) | 767 (64.02) |  |  |
| Not retired | 20,298 (43.68) | 431 (35.98) |  |  |
| **Type of study cohort** |  |  | 35.91 | <0.05 |
| Tracking | 18,924 (40.60) | 591 (49.21) |  |  |
| Comprehensive | 27,686 (59.40) | 610 (50.79) |  |  |
| **Ever exposed to SW** |  |  | 0.097 | 0.75 |
| Never exposed to SW(Daytime work only) | 37,433 (82.94) | 939 (82.59) |  |  |
| Ever exposed to SW | 7,701 (17.06) | 198 (17.41) |  |  |
| **SW exposure in longest job** |  |  | 2.72 | 0.26 |
| Not exposed to SW (Daytime work) | 37,392 (84.54) | 937 (83.51) |  |  |
| Night SW | 1,217 (2.75) | 26 (2.32) |  |  |
| Rotating SW | 5,622 (12.71) | 159 (14.17) |  |  |
| **SW exposure in current job** |  |  | 3.73 | 0.15 |
| Not exposed to SW (Daytime work) | 15,720 (86.86) | 307 (83.79) |  |  |
| Night SW | 601 (3.32) | 15 (4.08) |  |  |
| Rotating SW | 1,777 (9.82) | 46 (12.50) |  |  |
| **^a^** Reported frequencies are column percentages  **^b^** The statistic reported for all variables was the chi-square value  **^c^** Other included South Asian, Chinese, Filipino, Latin American, Japanese, Southeast Asian, Korean, Arab, West Asian, and Black.  CAD, Canadian dollars; BMI, body mass index; CES-D, the Center for Epidemiological Studies Depression Scale SW, shift work; REY, Rey auditory verbal learning; AF, Animal fluency; MAT, Mental alteration. | | | | |
