# Supplementary material for: The association between shift work exposure and cognitive impairment among middle-aged and older adults: Results from the Canadian Longitudinal Study on Aging (CLSA)
Source: PLoS One. 2023 Aug 23;18(8):e0289718. doi: 10.1371/journal.pone.0289718 (PMC10446236; doi:10.1371/journal.pone.0289718)
Supplement: S2 Appendix — (DOCX) [file pone.0289718.s003.docx]

**S2 Appendix**.

**Adjusted logistic regression models with estimates for all covariates**

| **Memory Function Impairment (REYI)** | | | | | | | | | | |  |
| --- | --- | --- | --- | --- | --- | --- | --- | --- | --- | --- | --- |
| **Adjusted logistic regression models [odds ratios (ORs) and 95% Confidence Intervals(CI)] for Memory Function Impairment (REYI)** | | | | | | | | | | |  |
|  | | **Model 1** | | | **Model 2** | | | **Model 3** | | |  |
|  | | **Ever exposed to SW** | | | **SW exposure in longest job** | | | **SW exposure in current job** | | |  |
|  | | Impaired cognition  OR (95% CI) ^a,b^ | | | Impaired cognition  OR (95% CI) ^a,b^ | | | Impaired cognition  OR (95% CI) ^a,b^ | | |  |
| **Ever exposed to SW** | |  | | |  | | |  | | |  |
| Never exposed to SW (Daytime work only) | | 1.00 | | | — | | | — | | |  |
| Ever exposed to SW | | 0.95 (0.81-1.11) | | | — | | | — | | |  |
| **SW exposure in longest job** | |  | | |  | | |  | | |  |
| Not exposed to SW  (Daytime work) | | — | | | 1.00 | | | — | | |  |
| Night SW | | — | | | 0.95 (0.67-1.33) | | | — | | |  |
| Rotating SW | | — | | | 0.97 (0.82-1.16) | | | — | | |  |
| **SW exposure in current job^c^** | |  | | |  | | |  | | |  |
| Not exposed to SW  (Daytime work) | | — | | | — | | | 1.00 | | |  |
| Night SW | | — | | | — | | | 1.47 (0.97-2.21) | | |  |
| Rotating SW | | — | | | — | | | 1.06 (0.79-1.41) | | |  |
| **Potential predictors** | |  | | |  | | |  | | |  |
| **Age (years)** | |  | | |  | | |  | | |  |
| 45-54 | | 1.00 | | | 1.00 | | | 1.00 | | |  |
| 55-64 | | **0.67 (0.57-0.78)*** | | | **0.67 (0.56-0.78)*** | | | **0.72 (0.60-0.87)*** | | |  |
| 65-74 | | **0.64 (0.52-0.80)*** | | | **0.65 (0.52-0.81)*** | | | **0.59 (0.38-0.93)*** | | |  |
| 75+ | | **0.72 (0.56-0.92)*** | | | **0.73 (0.57-0.93)*** | | | **0.39 (0.17-0.92)*** | | |  |
| **Sex** | |  | | |  | | |  | | |  |
| Male | | 1.00 | | | 1.00 | | | 1.00 | | |  |
| Female | | 0.93 (0.82-1.05) | | | 0.93 (0.82-1.05) | | | 1.05 (0.87-1.27) | | |  |
| **Ethnicity** | |  | | |  | | |  | | |  |
| Whites | | 1.00 | | | 1.00 | | | 1.00 | | |  |
| Other | | **1.89 (1.50-2.37)*** | | | **1.89 (1.50-2.38)*** | | | **2.12 (1.57-2.86)*** | | |  |
| **Marital status** | |  | | |  | | |  | | |  |
| With partner | | 1.00 | | | 1.00 | | | 1.00 | | |  |
| No partner | | 0.99 (0.85-1.14) | | | 0.98 (0.84-1.14) | | | 0.93 (0.75-1.15) | | |  |
| **Education level** | |  | | |  | | |  | | |  |
| Less than high school | | 1.00 | | | 1.00 | | | 1.00 | | |  |
| High school to some college | | 1.19 (0.97-1.47) | | | 1.18 (0.95-1.46) | | | 1.07 (0.71-1.61) | | |  |
| Bachelor’s degree and Higher | | **0.74 (0.58-0.93)*** | | | **0.73 (0.58-0.93)*** | | | 0.69 (0.45-1.05) | | |  |
| **Household income (CAD)** | |  | | |  | | |  | | |  |
| Less than $20,000 | | 1.00 | | | 1.00 | | | 1.00 | | |  |
| $20,000 or more, but less than $50,000 | | **0.53 (0.41-0.67)*** | | | **0.53 (0.41-0.67)*** | | | 0.81 (0.41-1.58) | | |  |
| $50,000 or more, but less than $100,000 | | **0.49 (0.38-0.63)*** | | | **0.50 (0.39-0.65)*** | | | 0.70 (0.36-1.36) | | |  |
| $100,000 and more | | **0.42 (0.32-0.55)*** | | | **0.42 (0.32-0.56)*** | | | 0.62 (0.32-1.19) | | |  |
| **Smoking** | |  | | |  | | |  | | |  |
| Never | | 1.00 | | | 1.00 | | | 1.00 | | |  |
| Former | | 1.12 (0.98-1.28) | | | 1.12 (0.98-1.28) | | | 1.17 (0.96-1.42) | | |  |
| Current | | 1.10 (0.89-1.35) | | | 1.10 (0.89-1.35) | | | 0.99 (0.73-1.35) | | |  |
| **Alcohol consumption** | |  | | |  | | |  | | |  |
| Never | | 1.00 | | | 1.00 | | | 1.00 | | |  |
| Drinks less than weekly | | 0.96 (0.80-1.14) | | | 0.94 (0.79-1.13) | | | 1.08 (0.82-1.44) | | |  |
| Drinks at least weekly | | **0.75 (0.63-0.89)*** | | | **0.73 (0.61-0.87)*** | | | **0.74 (0.57-0.97)*** | | |  |
| **BMI (kg/m^2^)** | |  | | |  | | |  | | |  |
| 20.0-24.99 (normal weight) | | 1.00 | | | 1.00 | | | 1.00 | | |  |
| <20.00 (underweight) | | 1.20 (0.86-1.68) | | | 1.25 (0.89-1.74) | | | 1.08 (0.63-1.83) | | |  |
| 25.0-29.99 (overweight) | | 1.06 (0.91-1.24) | | | 1.07 (0.92-1.25) | | | 1.18 (0.94-1.48) | | |  |
| >30.0 (obese) | | 0.92 (0.78-1.08) | | | 0.93 (0.78-1.10) | | | 1.00 (0.78-1.28) | | |  |
| **Depression** | |  | | |  | | |  | | |  |
| No (CES-D10 <10) | | 1.00 | | | 1.00 | | | 1.00 | | |  |
| Yes (CES-D10 ≥ 10) | | **1.31 (1.13-1.52)*** | | | **1.29 (1.10-150)*** | | | **1.30 (1.04-1.63)*** | | |  |
| **Multi-morbidity** | |  | | |  | | |  | | |  |
| Yes (≥2 chronic diseases) | | 1.00 | | | 1.00 | | | 1.00 | | |  |
| No (<2 chronic disease) | | 0.93 (0.81-1.07) | | | 0.94 (0.82-1.09) | | | 0.96 (0.77-1.19) | | |  |
| **Social Support Availability (SSA)** | |  | | |  | | |  | | |  |
| Low | | 1.00 | | | 1.00 | | | 1.00 | | |  |
| Medium | | **0.81 (0.70-0.94)*** | | | **0.81 (0.70-0.94)*** | | | 0.93 (0.74-1.15) | | |  |
| High | | **0.75 (0.65-0.88)*** | | | **0.75 (0.64-0.88)*** | | | **0.66 (0.53-0.82)*** | | |  |
| **Retirement status** | |  | | |  | | |  | | |  |
| Completely /partially retired | | 1.00 | | | 1.00 | | | — | | |  |
| Not retired | | 1.04 (0.88-1.23) | | | 1.04 (0.88-1.23) | | | — | | |  |
| **Type of study cohort** | |  | | |  | | |  | | |  |
| Tracking | | 1.00 | | | 1.00 | | | 1.00 | | |  |
| Comprehensive | | 1.11 (0.98-1.25) | | | 1.12 (0.99-1.27) | | | **1.28 (1.07-1.54)*** | | |  |
| ***** *P* value <0.05  ^a^ The ORs and 95% CI were calculated using survey analytical weights.  ^b^ Models are adjusted for age, sex, ethnicity, marital status, education, income, BMI, smoking, alcohol consumption, retirement status, depression , multi-morbidity, social support availability index , type of study cohort  ^c^ For current job, only those participants were included who reported currently working (not retired) (N=18,466),and the models are adjusted for all covariates mentioned above except for retirement status  SW, shift work; OR, odds ratio; CI, confidence interval. | | | | | | | | | | |  |
| **Memory Function Impairment (REYII)** | | | | | | | | | |  |  |
| **Adjusted logistic regression models [odds ratios (ORs) and 95% Confidence Intervals(CI)] for Memory Function Impairment (REYII)** | | | | | | | | | |  |  |
|  | **Model 1** | | | **Model 2** | | | **Model 3** | | |  |  |
|  | **Ever exposed to SW** | | | **SW exposure in longest job** | | | **SW exposure in current job** | | |  |  |
|  | Impaired cognition  OR (95% CI) ^a,b^ | | | Impaired cognition  OR (95% CI) ^a,b^ | | | Impaired cognition  OR (95% CI) ^a,b^ | | |  |  |
| **Ever exposed to SW** |  | | |  | | |  | | |  |  |
| Never exposed to SW (Daytime work only) | 1.00 | | | — | | | — | | |  |  |
| Ever exposed to SW | 1.08 (0.92-1.26) | | | — | | | — | | |  |  |
| **SW exposure in longest job** |  | | |  | | |  | | |  |  |
| Not exposed to SW  (Daytime work) | — | | | 1.00 | | | — | | |  |  |
| Night SW | — | | | **1.44 (1.03-2.01)*** | | | — | | |  |  |
| Rotating SW | — | | | 0.99 (0.82-1.16) | | | — | | |  |  |
| **SW exposure in current job^c^** |  | | |  | | |  | | |  |  |
| Not exposed to SW  (Daytime work) | — | | | — | | | 1.00 | | |  |  |
| Night SW | — | | | — | | | 1.28 (0.81-2.05) | | |  |  |
| Rotating SW | — | | | — | | | 1.03 (0.77-1.36) | | |  |  |
| **Potential predictors** |  | | |  | | |  | | |  |  |
| **Age (years)** |  | | |  | | |  | | |  |  |
| 45-54 | 1.00 | | | 1.00 | | | 1.00 | | |  |  |
| 55-64 | 0.88 (0.75-0.78) | | | 0.88 (0.75-1.03) | | | 0.91 (0.76-1.09) | | |  |  |
| 65-74 | 0.94 (0.76-1.16) | | | 0.94 (0.75-1.17) | | | 1.21 (0.77-1.90) | | |  |  |
| 75+ | **0.46 (0.35-0.59)*** | | | **0.46 (0.35-0.60)*** | | | **0.21 (0.62-0.68)*** | | |  |  |
| **Sex** |  | | |  | | |  | | |  |  |
| Male | 1.00 | | | 1.00 | | | 1.00 | | |  |  |
| Female | 1.03 (0.91-1.17) | | | 1.03 (0.90-1.16) | | | 0.90 (0.74-1.08) | | |  |  |
| **Ethnicity** |  | | |  | | |  | | |  |  |
| Whites | 1.00 | | | 1.00 | | | 1.00 | | |  |  |
| Other | **1.82 (1.41-2.35)*** | | | **1.75 (1.35-2.27)*** | | | **1.94 (1.36-2.76)*** | | |  |  |
| **Marital status** |  | | |  | | |  | | |  |  |
| With partner | 1.00 | | | 1.00 | | | 1.00 | | |  |  |
| No partner | 0.87 (0.74-1.02) | | | 0.87 (0.74-1.2) | | | 0.94 (0.74-1.20) | | |  |  |
| **Education level** |  | | |  | | |  | | |  |  |
| Less than high school | 1.00 | | | 1.00 | | | 1.00 | | |  |  |
| High school to some college | **1.52 (1.18-1.93)*** | | | **1.52 (1.19-1.96)*** | | | 1.44 (0.89-2.32) | | |  |  |
| Bachelor’s degree and Higher | 0.99 (0.76-1.30) | | | 1.01 (0.77-1.33) | | | 0.84 (0.51-1.39) | | |  |  |
| **Household income (CAD)** |  | | |  | | |  | | |  |  |
| Less than $20,000 | 1.00 | | | 1.00 | | | 1.00 | | |  |  |
| $20,000 or more, but less than $50,000 | **0.57 (0.44-0.74)*** | | | **0.57 (0.44-0.73)*** | | | 0.78 (0.38-1.59) | | |  |  |
| $50,000 or more, but less than $100,000 | **0.45 (0.35-0.60)*** | | | **0.44 (0.34-0.58)*** | | | 0.70 (0.35-1.42) | | |  |  |
| $100,000 and more | **0.44 (0.33-0.60)*** | | | **0.43 (0.32-0.58)*** | | | 0.74 (0.36-1.52) | | |  |  |
| **Smoking** |  | | |  | | |  | | |  |  |
| Never | 1.00 | | | 1.00 | | | 1.00 | | |  |  |
| Former | 1.01 (0.86-1.16) | | | 1.02 (0.89-1.17) | | | 1.08 (0.88-1.31) | | |  |  |
| Current | 1.16 (0.94-1.43) | | | 1.12 (0.90-1.38) | | | 1.14 (0.85-1.53) | | |  |  |
| **Alcohol consumption** |  | | |  | | |  | | |  |  |
| Never | 1.00 | | | 1.00 | | | 1.00 | | |  |  |
| Drinks less than weekly | 0.91 (0.75-1.09) | | | 0.92 (0.76-1.10) | | | 0.82 (0.60-1.10) | | |  |  |
| Drinks at least weekly | 0.90 (0.75-1.07) | | | 0.90 (0.75-1.07) | | | 0.74 (0.57-0.97) | | |  |  |
| **BMI (kg/m^2^)** |  | | |  | | |  | | |  |  |
| 20.0-24.99 (normal weight) | 1.00 | | | 1.00 | | | 1.00 | | |  |  |
| <20.00 (underweight) | 1.27 (0.86-1.86) | | | 1.37 (0.93-2.00) | | | **1.90 (1.12-3.22)*** | | |  |  |
| 25.0-29.99 (overweight) | 1.14 (0.98-1.32) | | | **1.20 (1.04-1.40)*** | | | 1.09 (0.88-1.36) | | |  |  |
| >30.0 (obese) | 1.12 (0.94-1.31) | | | 1.16 (0.98-1.37) | | | 1.13 (0.89-1.44) | | |  |  |
| **Depression** |  | | |  | | |  | | |  |  |
| No (CES-D10 <10) | 1.00 | | | 1.00 | | | 1.00 | | |  |  |
| Yes (CES-D10 ≥ 10) | **1.31 (1.11-1.53)*** | | | **1.31 (1.12-1.53)*** | | | 1.23 (0.97-1.57) | | |  |  |
| **Multi-morbidity** |  | | |  | | |  | | |  |  |
| Yes (≥2 chronic diseases) | 1.00 | | | 1.00 | | | 1.00 | | |  |  |
| No (<2 chronic disease) | 0.97 (0.84-1.12) | | | 1.00 (0.86-1.15) | | | 0.96 (0.75-1.22) | | |  |  |
| **Social Support Availability (SSA)** |  | | |  | | |  | | |  |  |
| Low | 1.00 | | | 1.00 | | | 1.00 | | |  |  |
| Medium | 0.89 (0.77-1.04) | | | 0.90 (0.70-0.94) | | | 1.03 (0.83-1.28) | | |  |  |
| High | 0.95 (0.82-1.11) | | | 0.96 (0.64-0.88) | | | 0.66 (0.53-1.22) | | |  |  |
| **Retirement status** |  | | |  | | |  | | |  |  |
| Completely /partially retired | 1.00 | | | 1.00 | | | — | | |  |  |
| Not retired | 1.13 (0.96-1.33) | | | 1.14 (0.96-1.34) | | | — | | |  |  |
| **Type of study cohort** |  | | |  | | |  | | |  |  |
| Tracking | 1.00 | | | 1.00 | | | 1.00 | | |  |  |
| Comprehensive | 1.06 (0.94-1.21) | | | 1.07 (0.94-1.22) | | | 1.08 (0.89-1.30) | | |  |  |
| ***** *P* value <0.05  ^a^ The ORs and 95% CI were calculated using survey analytical weights.  ^b^ Models are adjusted for age, sex, ethnicity, marital status, education, income, BMI, smoking, alcohol consumption, retirement status, depression , multi-morbidity, social support availability index , type of study cohort  ^c^ For current job, only those participants were included who reported currently working (not retired) (N=18,466),and the models are adjusted for all covariates mentioned above except for retirement status  SW, shift work; OR, odds ratio; CI, confidence interval. | | | | | | | | | |  |  |
| **Executive Function Impairment (AF2)** | | | | | | | | | | |  |
| **Adjusted logistic regression models [odds ratios (ORs) and 95% Confidence Intervals(CI)] for Executive Function Impairment (AF2)** | | | | | | | | | | |  |
|  | | **Model 1** | | | **Model 2** | | | **Model 3** | | |  |
|  | | **Ever exposed to SW** | | | **SW exposure in longest job** | | | **SW exposure in current job** | | |  |
|  | | Impaired cognition  OR (95% CI) ^a,b^ | | | Impaired cognition  OR (95% CI) ^a,b^ | | | Impaired cognition  OR (95% CI) ^a,b^ | | |  |
| **Ever exposed to SW** | |  | | |  | | |  | | |  |
| Never exposed to SW (Daytime work only) | | 1.00 | | | — | | | — | | |  |
| Ever exposed to SW | | 1.12 (0.96-1.30) | | | — | | | — | | |  |
| **SW exposure in longest job** | |  | | |  | | |  | | |  |
| Not exposed to SW  (Daytime work) | | — | | | 1.00 | | | — | | |  |
| Night SW | | — | | | 1.27 (0.92-1.76) | | | — | | |  |
| Rotating SW | | — | | | 1.07 (0.90-1.26) | | | — | | |  |
| **SW exposure in current job^c^** | |  | | |  | | |  | | |  |
| Not exposed to SW  (Daytime work) | | — | | | — | | | 1.00 | | |  |
| Night SW | | — | | | — | | | 1.32 (0.87-1.99) | | |  |
| Rotating SW | | — | | | — | | | 0.95 (0.73-1.26) | | |  |
| **Potential predictors** | |  | | |  | | |  | | |  |
| **Age (years)** | |  | | |  | | |  | | |  |
| 45-54 | | 1.00 | | | 1.00 | | | 1.00 | | |  |
| 55-64 | | **0.65 (0.56-0.75)*** | | | **0.64 (0.55-0.75)*** | | | **0.63 (0.52-0.76)*** | | |  |
| 65-74 | | **0.56 (0.45-0.70)*** | | | **0.57 (0.45-0.71)*** | | | **0.45 (0.28-0.69)*** | | |  |
| 75+ | | **0.51 (0.39-0.67)*** | | | **0.51 (0.39-0.67)*** | | | **0.27 (0.12-0.63)*** | | |  |
| **Sex** | |  | | |  | | |  | | |  |
| Male | | 1.00 | | | 1.00 | | | 1.00 | | |  |
| Female | | 0.98 (0.86-1.12) | | | 0.97 (0.85-1.11) | | | 1.10 (0.92-1.33) | | |  |
| **Ethnicity** | |  | | |  | | |  | | |  |
| Whites | | 1.00 | | | 1.00 | | | 1.00 | | |  |
| Other | | **3.27 (2.70-3.98)*** | | | **3.30 (2.72-4.01)*** | | | **4.24 (3.32-5.40)*** | | |  |
| **Marital status** | |  | | |  | | |  | | |  |
| With partner | | 1.00 | | | 1.00 | | | 1.00 | | |  |
| No partner | | 0.92 (0.79-1.07) | | | 0.91 (0.78-1.06) | | | 0.91 (0.72-1.15) | | |  |
| **Education level** | |  | | |  | | |  | | |  |
| Less than high school | | 1.00 | | | 1.00 | | | 1.00 | | |  |
| High school to some college | | **1.53 (1.18-1.97)*** | | | **1.52 (1.17-1.97)*** | | | 1.84 (0.71-1.61) | | |  |
| Bachelor’s degree and Higher | | 0.90 (0.68-1.18) | | | 0.89 (0.67-1.18) | | | 1.02 (0.58-1.80) | | |  |
| **Household income (CAD)** | |  | | |  | | |  | | |  |
| Less than $20,000 | | 1.00 | | | 1.00 | | | 1.00 | | |  |
| $20,000 or more, but less than $50,000 | | 0.81 (0.63-1.03) | | | 0.85 (0.66-1.11) | | | 0.73 (0.42-1.28) | | |  |
| $50,000 or more, but less than $100,000 | | **0.64 (0.50-0.83)*** | | | **0.65 (0.51-0.86)*** | | | 0.64 (0.38-1.08) | | |  |
| $100,000 and more | | **0.47 (0.36-0.62)*** | | | **0.48 (0.36-0.63)*** | | | **0.45 (0.26-0.76)*** | | |  |
| **Smoking** | |  | | |  | | |  | | |  |
| Never | | 1.00 | | | 1.00 | | | 1.00 | | |  |
| Former | | 1.05 (0.91-1.19) | | | 1.05 (0.92-1.20) | | | 1.13 (0.94-1.36) | | |  |
| Current | | 1.04 (0.84-1.28) | | | 0.99 (0.80-1.23) | | | 1.23 (0.92-1.65) | | |  |
| **Alcohol consumption** | |  | | |  | | |  | | |  |
| Never | | 1.00 | | | 1.00 | | | 1.00 | | |  |
| Drinks less than weekly | | 0.84 (0.70-1.00) | | | 0.85 (0.71-1.02) | | | 1.03 (0.78-1.35) | | |  |
| Drinks at least weekly | | **0.78 (0.66-0.92)*** | | | **0.78 (0.66-0.93)*** | | | 0.96 (0.74-1.23) | | |  |
| **BMI (kg/m^2^)** | |  | | |  | | |  | | |  |
| 20.0-24.99 (normal weight) | | 1.00 | | | 1.00 | | | 1.00 | | |  |
| <20.00 (underweight) | | 0.96 (0.65-1.41) | | | 0.97 (0.65-1.43) | | | 1.06 (0.63-1.76) | | |  |
| 25.0-29.99 (overweight) | | 1.13 (0.97-1.32) | | | 1.01 (0.95-1.30) | | | 1.16 (0.94-1.43) | | |  |
| >30.0 (obese) | | 0.97 (0.82-1.15) | | | 0.98 (0.83-1.17) | | | 0.95 (0.78-1.20) | | |  |
| **Depression** | |  | | |  | | |  | | |  |
| No (CES-D10 <10) | | 1.00 | | | 1.00 | | | 1.00 | | |  |
| Yes (CES-D10 ≥ 10) | | 1.15 (0.98-1.34) | | | 1.13 (0.96-1.32) | | | 1.17 (0.94-1.46) | | |  |
| **Multi-morbidity** | |  | | |  | | |  | | |  |
| Yes (≥2 chronic diseases) | | 1.00 | | | 1.00 | | | 1.00 | | |  |
| No (<2 chronic disease) | | 1.06 (0.92-1.24) | | | 1.07 (0.91-1.25) | | | 1.17 (0.90-1.50) | | |  |
| **Social Support Availability (SSA)** | |  | | |  | | |  | | |  |
| Low | | 1.00 | | | 1.00 | | | 1.00 | | |  |
| Medium | | **0.85 (0.73-0.98)*** | | | **0.84 (0.73-0.97)*** | | | 0.89 (0.72-1.09) | | |  |
| High | | **0.84 (0.71-0.99)*** | | | **0.83 (0.70-0.98)*** | | | 0.87 (0.68-1.06) | | |  |
| **Retirement status** | |  | | |  | | |  | | |  |
| Completely /partially retired | | 1.00 | | | 1.00 | | | — | | |  |
| Not retired | | 1.09 (0.93-1.27) | | | 1.10 (0.93-1.25) | | | — | | |  |
| **Type of study cohort** | |  | | |  | | |  | | |  |
| Tracking | | 1.00 | | | 1.00 | | | 1.00 | | |  |
| Comprehensive | | 1.03 (0.91-1.17) | | | 1.04 (0.91-1.18) | | | 1.07 (0.89-1.28) | | |  |
| ***** *P* value <0.05  ^a^ The ORs and 95% CI were calculated using survey analytical weights.  ^b^ Models are adjusted for age, sex, ethnicity, marital status, education, income, BMI, smoking, alcohol consumption, retirement status, depression , multi-morbidity, social support availability index , type of study cohort  ^c^ For current job, only those participants were included who reported currently working (not retired) (N=18,466),and the models are adjusted for all covariates mentioned above except for retirement status  SW, shift work; OR, odds ratio; CI, confidence interval. | | | | | | | | | | |  |
| **Executive Function Impairment (MAT)** | | | | | | | | | | | |
| **Adjusted logistic regression models [odds ratios (ORs) and 95% Confidence Intervals(CI)] for Executive Function Impairment (MAT)** | | | | | | | | | | | |
|  | | | **Model 1** | | | **Model 2** | | | **Model 3** | | |
|  | | | **Ever exposed to SW** | | | **SW exposure in longest job** | | | **SW exposure in current job** | | |
|  | | | Impaired cognition  OR (95% CI) ^a,b^ | | | Impaired cognition  OR (95% CI) ^a,b^ | | | Impaired cognition  OR (95% CI) ^a,b^ | | |
| **Ever exposed to SW** | | |  | | |  | | |  | | |
| Never exposed to SW (Daytime work only) | | | 1.00 | | | — | | | — | | |
| Ever exposed to SW | | | **1.14 (1.00-1.29)*** | | | — | | | — | | |
| **SW exposure in longest job** | | |  | | |  | | |  | | |
| Not exposed to SW  (Daytime work) | | | — | | | 1.00 | | | — | | |
| Night SW | | | — | | | 1.20 (0.90-1.60) | | | — | | |
| Rotating SW | | | — | | | **1.16 (1.01-1.34)*** | | | — | | |
| **SW exposure in current job^c^** | | |  | | |  | | |  | | |
| Not exposed to SW  (Daytime work) | | | — | | | — | | | 1.00 | | |
| Night SW | | | — | | | — | | | 1.31 (0.87-1.99) | | |
| Rotating SW | | | — | | | — | | | **1.36 (1.06-1.74)*** | | |
| **Potential predictors** | | |  | | |  | | |  | | |
| **Age (years)** | | |  | | |  | | |  | | |
| 45-54 | | | 1.00 | | | 1.00 | | | 1.00 | | |
| 55-64 | | | **0.73 (0.63-0.84)*** | | | **0.73 (0.63-0.84)*** | | | **0.76 (0.64-0.91)*** | | |
| 65-74 | | | **0.74 (0.61-0.89)*** | | | **0.73 (0.60-0.87)*** | | | **0.61 (0.41-0.91)*** | | |
| 75+ | | | 0.87 (0.70-1.08) | | | 0.86 (0.69-1.07) | | | 0.58 (0.28-1.20) | | |
| **Sex** | | |  | | |  | | |  | | |
| Male | | | 1.00 | | | 1.00 | | | 1.00 | | |
| Female | | | 1.03 (0.92-1.16) | | | 1.03 (0.93-1.17) | | | 1.07 (0.91-1.27) | | |
| **Ethnicity** | | |  | | |  | | |  | | |
| Whites | | | 1.00 | | | 1.00 | | | 1.00 | | |
| Other | | | **2.77 (2.28-3.36)*** | | | **2.79 (2.30-3.40)*** | | | **3.06 (2.39-3.93)*** | | |
| **Marital status** | | |  | | |  | | |  | | |
| With partner | | | 1.00 | | | 1.00 | | | 1.00 | | |
| No partner | | | 0.88 (0.76-1.01) | | | 0.89 (0.77-1.02) | | | **0.70 (0.56-0.88)*** | | |
| **Education level** | | |  | | |  | | |  | | |
| Less than high school | | | 1.00 | | | 1.00 | | | 1.00 | | |
| High school to some college | | | **1.23 (1.03-1.49)*** | | | **1.26 (1.04-1.52)*** | | | **1.54 (1.03-2.32)*** | | |
| Bachelor’s degree and Higher | | | **0.76 (0.61-0.93)*** | | | **0.76 (0.62-0.94)*** | | | 1.14 (0.75-1.72) | | |
| **Household income (CAD)** | | |  | | |  | | |  | | |
| Less than $20,000 | | | 1.00 | | | 1.00 | | | 1.00 | | |
| $20,000 or more, but less than $50,000 | | | **0.66 (0.53-0.83)*** | | | **0.67 (0.54-0.83)*** | | | 0.91 (0.52-1.57) | | |
| $50,000 or more, but less than $100,000 | | | **0.46 (0.37-0.58)*** | | | **0.47 (0.38-0.59)*** | | | **0.57 (0.34-0.98)*** | | |
| $100,000 and more | | | **0.34 (0.26-0.43)*** | | | **0.34 (0.27-0.44)*** | | | **0.37 (0.21-0.64)*** | | |
| **Smoking** | | |  | | |  | | |  | | |
| Never | | | 1.00 | | | 1.00 | | | 1.00 | | |
| Former | | | 1.01 (0.89-1.14) | | | 1.01 (0.90-1.15) | | | 1.11 (0.94-1.33) | | |
| Current | | | **1.34 (1.12-1.61)*** | | | **1.30 (1.08-1.57)*** | | | **1.34 (1.03-1.74)*** | | |
| **Alcohol consumption** | | |  | | |  | | |  | | |
| Never | | | 1.00 | | | 1.00 | | | 1.00 | | |
| Drinks less than weekly | | | 1.02 (0.87-1.19) | | | 1.03 (0.88-1.22) | | | 1.09 (0.84-1.43) | | |
| Drinks at least weekly | | | 1.01 (0.86-1.19) | | | 1.02 (0.87-1.20) | | | 1.16 (0.90-1.50) | | |
| **BMI (kg/m^2^)** | | |  | | |  | | |  | | |
| 20.0-24.99 (normal weight) | | | 1.00 | | | 1.00 | | | 1.00 | | |
| <20.00 (underweight) | | | **1.63 (1.20-2.19)*** | | | **1.60 (1.18-2.17)*** | | | 1.18 (0.70-1.97) | | |
| 25.0-29.99 (overweight) | | | 1.13 (0.98-1.29) | | | **1.14 (0.99-1.30)*** | | | 1.20 (0.98-1.45) | | |
| >30.0 (obese) | | | **1.23 (1.06-1.43)*** | | | **1.23 (1.06-1.43)*** | | | **1.35 (1.08-1.68)*** | | |
| **Depression** | | |  | | |  | | |  | | |
| No (CES-D10 <10) | | | 1.00 | | | 1.00 | | | 1.00 | | |
| Yes (CES-D10 ≥ 10) | | | **1.29 (1.123-1.48)*** | | | **1.31 (1.14-1.50)*** | | | **1.34 (1.10-1.64)*** | | |
| **Multi-morbidity** | | |  | | |  | | |  | | |
| Yes (≥2 chronic diseases) | | | 1.00 | | | 1.00 | | | 1.00 | | |
| No (<2 chronic disease) | | | 0.93 (0.82-1.04) | | | 0.94 (0.83-1.07) | | | 1.06 (0.86-1.32) | | |
| **Social Support Availability (SSA)** | | |  | | |  | | |  | | |
| Low | | | 1.00 | | | 1.00 | | | 1.00 | | |
| Medium | | | 0.94 (0.83-1.08) | | | 0.95 (0.83-1.08) | | | 0.89 (0.72-1.08) | | |
| High | | | 0.98 (0.85-1.13) | | | 0.98 (0.84-1.13) | | | 0.87 (0.68-1.06) | | |
| **Retirement status** | | |  | | |  | | |  | | |
| Completely /partially retired | | | 1.00 | | | 1.00 | | | — | | |
| Not retired | | | 1.02 (0.89-1.19) | | | 1.02 (0.89-1.18) | | | — | | |
| **Type of study cohort** | | |  | | |  | | |  | | |
| Tracking | | | 1.00 | | | 1.00 | | | 1.00 | | |
| Comprehensive | | | 0.97 (0.87-1.09) | | | 0.98 (0.88-1.10) | | | 1.03 (0.87-1.22) | | |
| ***** *P* value <0.05  ^a^ The ORs and 95% CI were calculated using survey analytical weights.  ^b^ Models are adjusted for age, sex, ethnicity, marital status, education, income, BMI, smoking, alcohol consumption, retirement status, depression , multi-morbidity, social support availability index , type of study cohort  ^c^ For current job, only those participants were included who reported currently working (not retired) (N=18,466),and the models are adjusted for all covariates mentioned above except for retirement status  SW, shift work; OR, odds ratio; CI, confidence interval. | | | | | | | | | | | |

| **Overall cognitive impairment** | | | |
| --- | --- | --- | --- |
| **Adjusted logistic regression models [odds ratios (ORs) and 95% Confidence Intervals(CI)] for Overall cognitive impairment** | | | |
|  | **Model 1** | **Model 2** | **Model 3** |
|  | **Ever exposed to SW** | **SW exposure in longest job** | **SW exposure in current job** |
|  | Impaired cognition  OR (95% CI) ^a,b^ | Impaired cognition  OR (95% CI) ^a,b^ | Impaired cognition  OR (95% CI) ^a,b^ |
| **Ever exposed to SW** |  |  |  |
| Never exposed to SW (Daytime work only) | 1.00 | — | — |
| Ever exposed to SW | 1.12 (0.92-1.35) | — | — |
| **SW exposure in longest job** |  |  |  |
| Not exposed to SW  (Daytime work) | — | 1.00 | — |
| Night SW | — | **1.53 (1.04-2.26)*** | — |
| Rotating SW | — | 1.02 (0.83-1.27) | — |
| **SW exposure in current job^c^** |  |  |  |
| Not exposed to SW  (Daytime work) | — | — | 1.00 |
| Night SW | — | — | **1.79 (1.08-2.96)*** |
| Rotating SW | — | — | 1.04 (0.73-1.17) |
| **Potential predictors** |  |  |  |
| **Age (years)** |  |  |  |
| 45-54 | 1.00 | 1.00 | 1.00 |
| 55-64 | **0.63 (0.52-0.77)*** | **0.63 (0.51-0.76)*** | **0.65 (0.51-0.83)*** |
| 65-74 | **0.58 (0.44-0.77)*** | **0.58 (0.44-0.77)*** | 0.82 (0.48-1.43) |
| 75+ | **0.44 (0.31-0.62)*** | **0.44 (0.31-0.63)*** | 0.66 (0.24-1.86) |
| **Sex** |  |  |  |
| Male | 1.00 | 1.00 | 1.00 |
| Female | 0.89 (0.76-1.05) | 0.89 (0.75-1.04) | 0.93 (0.73-1.17) |
| **Ethnicity** |  |  |  |
| Whites | 1.00 | 1.00 | 1.00 |
| Other | **3.88 (3.04-4.94)*** | **3.88 (3.05-4.94)*** | **4.83 (3.55-6.57)*** |
| **Marital status** |  |  |  |
| With partner | 1.00 | 1.00 | 1.00 |
| No partner | 0.87 (0.73-1.06) | 0.90 (0.74-1.07) | 0.80 (0.60-1.06) |
| **Education level** |  |  |  |
| Less than high school | 1.00 | 1.00 | 1.00 |
| High school to some college | **2.26 (1.63-3.13)*** | **2.37 (1.69-3.32)*** | **2.15 (1.17-3.98)*** |
| Bachelor’s degree and Higher | 1.06 (0.73-1.52) | 1.11 (0.78-1.61) | 0.98 (0.51-1.88) |
| **Household income (CAD)** |  |  |  |
| Less than $20,000 | 1.00 | 1.00 | 1.00 |
| $20,000 or more, but less than $50,000 | **0.45 (0.34-0.58)*** | **0.48 (0.37-0.63)*** | 0.59 (0.32-1.16) |
| $50,000 or more, but less than $100,000 | **0.32 (0.24-0.43)*** | **0.34 (0.25-0.45)*** | **0.44 (0.23-0.84)*** |
| $100,000 and more | **0.22 (0.16-0.31)*** | **0.23 (0.16-0.32)*** | **0.30 (0.15-0.58)*** |
| **Smoking** |  |  |  |
| Never | 1.00 | 1.00 | 1.00 |
| Former | 0.99 (0.84-1.19) | 1.00 (0.84-1.19) | 1.01 (0.78-1.30) |
| Current | 1.25 (0.98-1.60) | 1.20 (0.94-1.54) | 1.23 (0.87-1.76) |
| **Alcohol consumption** |  |  |  |
| Never | 1.00 | 1.00 | 1.00 |
| Drinks less than weekly | 0.84 (0.68-1.04) | 0.87 (0.70-1.07) | 0.89 (0.63-1.24) |
| Drinks at least weekly | **0.72 (0.59-0.89)*** | **0.74 (0.59-0.91)*** | 0.81 (0.58-1.12) |
| **BMI (kg/m^2^)** |  |  |  |
| 20.0-24.99 (normal weight) | 1.00 | 1.00 | 1.00 |
| <20.00 (underweight) | 1.48 (0.95-2.27) | **1.60 (1.18-2.17)*** | 1.33 (0.70-2.54) |
| 25.0-29.99 (overweight) | 1.10 (0.90-1.34) | 1.14 (0.99-1.30) | 1.21 (0.92-1.60) |
| >30.0 (obese) | 1.10 (0.88-1.37) | 1.23 (1.06-1.43) | 1.25 (0.92-1.71) |
| **Depression** |  |  |  |
| No (CES-D10 <10) | 1.00 | 1.00 | 1.00 |
| Yes (CES-D10 ≥ 10) | **1.55 (1.29-1.86)*** | **1.53 (1.28-1.84)*** | **1.80 (1.38-2.34)*** |
| **Multi-morbidity** |  |  |  |
| Yes (≥2 chronic diseases) | 1.00 | 1.00 | 1.00 |
| No (<2 chronic disease) | 0.97 (0.80-1.17) | 0.99 (0.82-1.20) | 1.22 (0.91-1.64) |
| **Social Support Availability (SSA)** |  |  |  |
| Low | 1.00 | 1.00 | 1.00 |
| Medium | **0.80 (0.67-0.97)*** | **0.81 (0.67-0.97)*** | 0.91 (0.69-1.20) |
| High | 0.90 (0.74-1.10) | 0.93 (0.76-1.13) | 0.89 (0.67-1.19) |
| **Retirement status** |  |  |  |
| Completely /partially retired | 1.00 | 1.00 | — |
| Not retired | 1.07 (0.87-1.32) | 1.08 (0.88-1.34) | — |
| **Type of study cohort** |  |  |  |
| Tracking | 1.00 | 1.00 | 1.00 |
| Comprehensive | 1.01 (0.86-1.19) | 1.03 (0.88-1.21) | 1.12 (0.88-1.42) |
| ***** *P* value <0.05  ^a^ The ORs and 95% CI were calculated using survey analytical weights.  ^b^ Models are adjusted for age, sex, ethnicity, marital status, education, income, BMI, smoking, alcohol consumption, retirement status, depression , multi-morbidity, social support availability index , type of study cohort  ^c^ For current job, only those participants were included who reported currently working (not retired) (N=18,466),and the models are adjusted for all covariates mentioned above except for retirement status  SW, shift work; OR, odds ratio; CI, confidence interval. | | | |
