# Supplementary material for: The association between shift work exposure and cognitive impairment among middle-aged and older adults: Results from the Canadian Longitudinal Study on Aging (CLSA)
Source: PLoS One. 2023 Aug 23;18(8):e0289718. doi: 10.1371/journal.pone.0289718 (PMC10446236; doi:10.1371/journal.pone.0289718)
Supplement: Attachment — Submitted filename: PONE-D-23-11981.docx [file pone.0289718.s004.docx]

**Peer Review - PONE-D-23-11981**

This is a very interesting and succinct secondary analysis exploring the association between shift work and cognitive impairment using data from the CLSA. Below are some suggestions to improve the manuscript.

1. The theoretical foundations and mechanisms linking shift work and cognitive impairment should be laid out and described in the introduction. I observe that the authors do mention these mechanisms in various places in the manuscript (e.g., desynchronization of the circadian rhythm on p. 3), though readers may find it helpful to have the theory laid out at the beginning of the manuscript. The authors may then circle back in the discussion to identify which theory or mechanism appears to be most supported by the results.

2. The literature review could benefit from more detail regarding the characteristics of the published studies. For example, what were the age ranges of participants in these studies, how was cognition measured, what were the sample sizes, were these studies cross-sectional or longitudinal, etc.? These additional details could help the authors identify reasons for discrepancies between studies and set the stage for them to more succinctly explain the novelty of their work and its contribution to the literature.

3. The authors combined Tracking and Comprehensive Cohort participants into a single analytical sample. However, did the authors conduct a preliminary assessment to verify the validity of joining the cohorts? This issue is most important for the cognitive tests, which can be influenced by the mode of administration (in-person versus telephone). The authors should check whether the distribution of cognitive test scores was similar across both cohorts and report this information in an appendix.

4. The authors should specify that they conducted a complete case analysis. The authors should also examine the potential impact of missing data on their results. For example, were the mean z-scores lower for persons with missing shift work data compared to those with complete shift work data? Was the proportion of people reporting shift work different among participants with missing versus complete cognition data? These issues should be addressed in the analysis to provide some indication as to whether missing data may have biased the results.

5. The CLSA is subject to recruitment bias on cognition because persons with overt cognitive impairment were excluded from the study. Additionally, volunteer bias may also be an issue, as the most cognitively healthy subgroup of the population may have chosen to participate. This is probably why the overall proportion of participants with cognitive impairment decreases as age group increases (Table 2). The authors should discuss how the recruitment of a cognitively healthy sample might bias their results (toward or away from the null).

6. The data in CLSA did not permit the authors to assess the length of exposure to shift work. Please explain how this limitation may have biased the findings.

7. To encourage transparency in the reporting of research results, I strongly recommend the authors report their study in line with the STROBE checklist (<https://www.strobe-statement.org/checklists/>) and include a copy of the completed checklist as an appendix.

8. p. 4, lines 74-75: Comprehensive Cohort participants also provided data during a site visit at their local data collection site. In fact, the entire neuropsychological battery was administered to Comprehensive participants at the data collection site.

9. p. 7, line 136: please cite your justifications for including the specific set of covariates described in ‘potential predictors’. Provide the citations that led you to choose these covariates over others.

10. p. 11, line 180: change ‘high odds’ to ‘higher odds’.

11. p. 15, line 247: Please verify whether reference # 55 should actually be reference # 21.

12. The CLSA-mandated acknowledgement is not complete. Please check with CLSA and obtain the complete acknowledgement.
